# Central Odontogenic Fibroma of the Mandible: A Case Report

**DOI:** 10.7759/cureus.99735

**Published:** 2025-12-20

**Authors:** Nivetha D, Christeffi Mabel, Abimathi R, Chamundeeswari P, Sakthi Sreedevi

**Affiliations:** 1 Oral Medicine and Radiology, Chettinad Dental College and Research Institute, Chennai, IND

**Keywords:** case report, central odontogenic fibroma, cof, mixed lesion of mandible, radiolucent lesion

## Abstract

Central odontogenic fibroma (COF) is a rare neoplasm, diagnostically challenging and critical to evaluate the lesion's clinical, radiographic and histological studies. Hereby we report a 19-year-old male patient with an initial complaint of pain in the left side of his face which on intraoral clinical examination revealed edentulous 47 region and on radiological investigation presented as well defined radiolucent lesion encircling the impacted 47 with a single speck of radiopacity within the radiolucency following which the lesion was excised along with extraction of impacted tooth and diagnosed histopathologically as COF. Although the patient’s primary manifestation was temporomandibular joint disorder (TMD), the diagnosis of COF brought attention to the importance of thorough evaluation. This case underscores the diagnostic challenge posed by a mixed presentation involving both COF and overlapping temporomandibular joint-related symptoms, highlighting the need for careful clinical-radiological correlation to avoid misdiagnosis and ensure timely, appropriate management.

## Introduction

The WHO in 2005 classified odontogenic fibroma as rare benign odontogenic tumor of mesenchymal origin, with International Classification of Diseases code 9321/0 [1]. From 2017 onward, the WHO categorized the lesion by its anatomical site (central vs. peripheral) instead of relying on histological criteria [2]. Of all the odontogenic tumors of the jaws, central odontogenic fibroma (COF) accounts for 0.1% [3]. According to the WHO, central odontogenic fibroma is classified into two variants: an epithelial-rich (WHO) type and an epithelial-poor (simple) type [4]. COF has been reported across a broad age range, with a noted female predilection [5]. In the maxilla, the lesion typically involves the anterior segment, while in the mandible it commonly involves the premolar-molar region [5,6]. The majority of central odontogenic fibromas are unilocular radiolucent lesions with well-defined borders, but they may also appear as multilocular lesions and in rare instances may exhibit a mixed radiolucent/radiopaque appearance with poorly defined or diffused borders [7]. The mixed stage can be due to internal calcification and necessitate histopathological confirmation. The great variability in radiological appearance of the central odontogenic fibroma often makes routine imaging insufficient for narrowing the differential diagnosis. Although conventional radiographs are useful, cone beam computed tomography (CBCT) has become an important adjunct for three‑dimensional assessment of odontogenic lesions and impacted teeth, improving localization and surgical planning [8].

## Case presentation

A 19-year-old male patient reported to the department with a chief complaint of pain in his left lower face for the past five days. He also gives a history of locking of the jaw while opening for the past two months which reduces by its own. No relevant medical and dental history reported.

**Figure 1:**
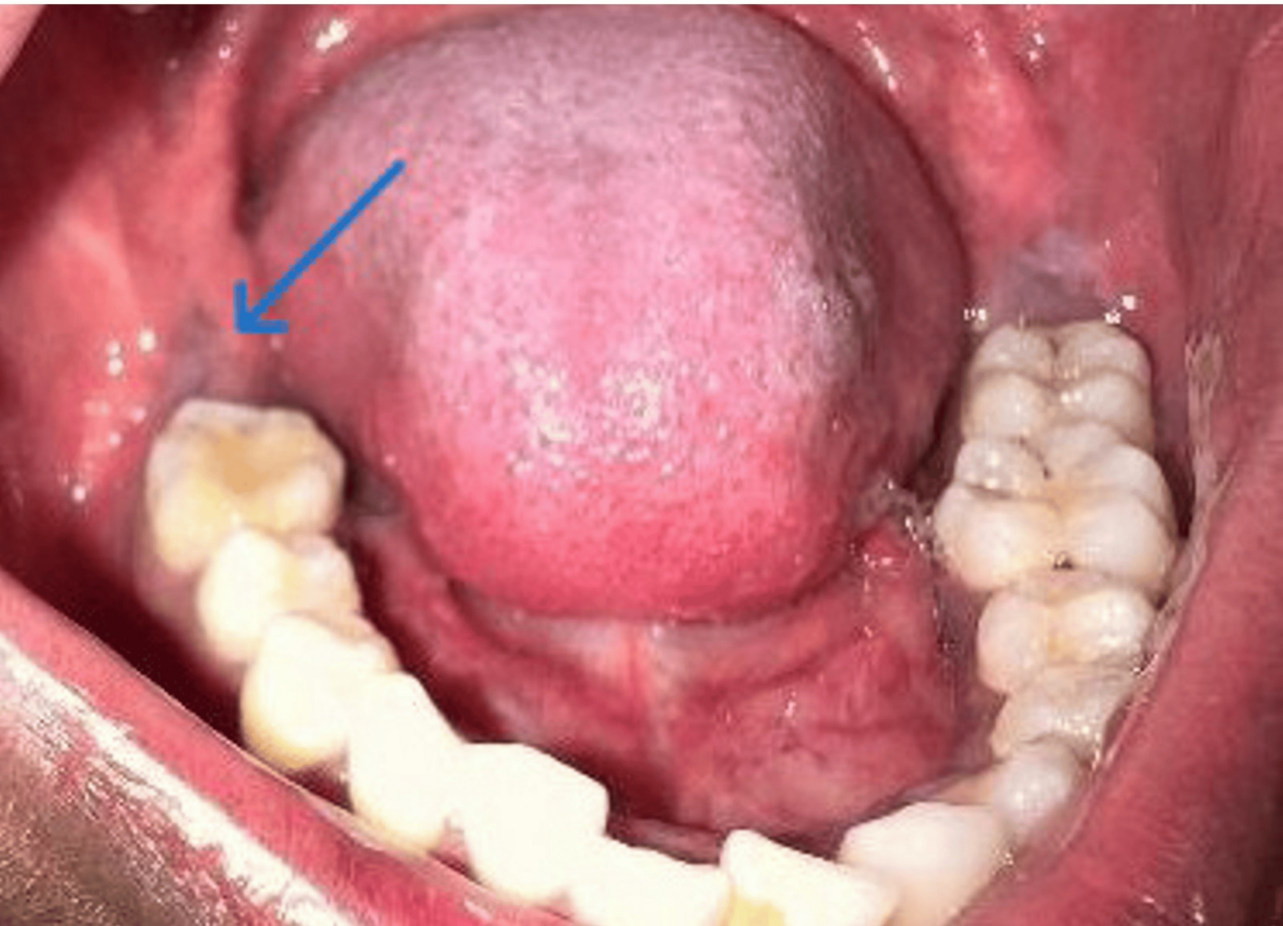
Intraoral image showing unerupted 47 Preoperative intraoral clinical photograph showing normal oral mucosa overlying the region of the unerupted mandibular right second molar (47) (blue arrow), with no visible mucosal abnormality.

On temporomandibular joint (TMJ) examination, tenderness was noted in the left preauricular region during opening and the jaw was deviating to the right side. Masticatory muscles were tender on the left side with a normal interincisal opening of 40 mm. Considering the above history and the clinical examination, a provisional diagnosis of TMJ internal derangement with reduction with intermittent locking in left side was given. On further evaluation, intraoral examination revealed unerupted 47 (Figure 1) with no evidence of swelling. An orthopantomogram (OPG) was taken as part of the TMJ assessment.

**Figure 2:**
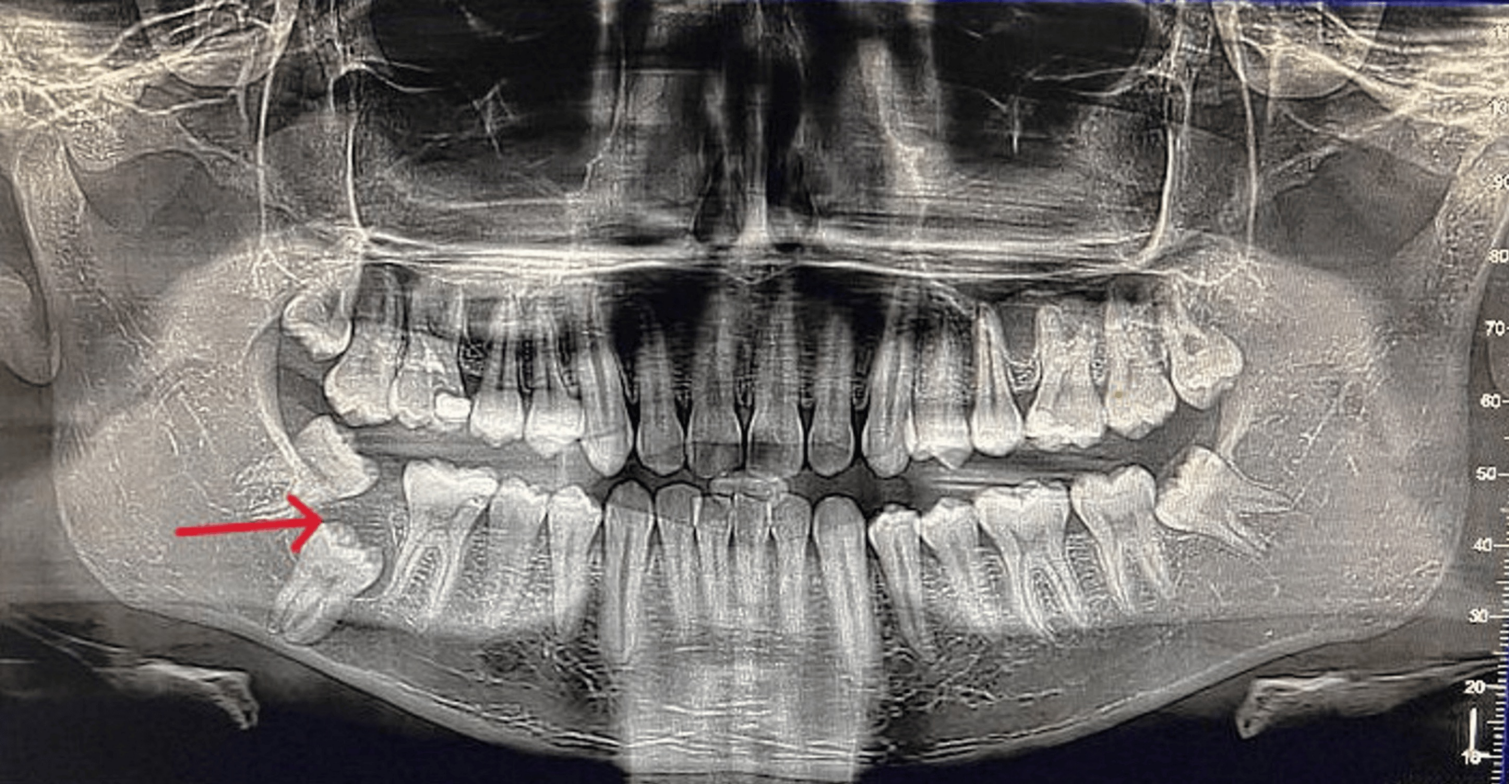
Orthopantomogram (OPG) showing impacted 47 with lesion Orthopantomogram demonstrating an unerupted mandibular right second molar (47) with a well-defined radiolucency surrounding the coronal portion of the tooth (red arrow). The radiolucent area appears unilocular with a corticated margin and is confined to the crown region of 47.

In OPG, no abnormalities were noted in the TMJ. However, it incidentally revealed impacted 47 with a well-defined unilocular radiolucency and a sclerotic border in the body of the mandible coronal to the 47 region, which measured about 1*1 cm in size approximately, encircling the cemento-enamel junction (CEJ) of 47 (Figure 2). CBCT was performed to assess impacted 47 and inferior alveolar nerve canal. For further assessment of the TMJ symptoms, Magnetic Resonance Imaging (MRI) was performed concurrently.

**Figure 3:**
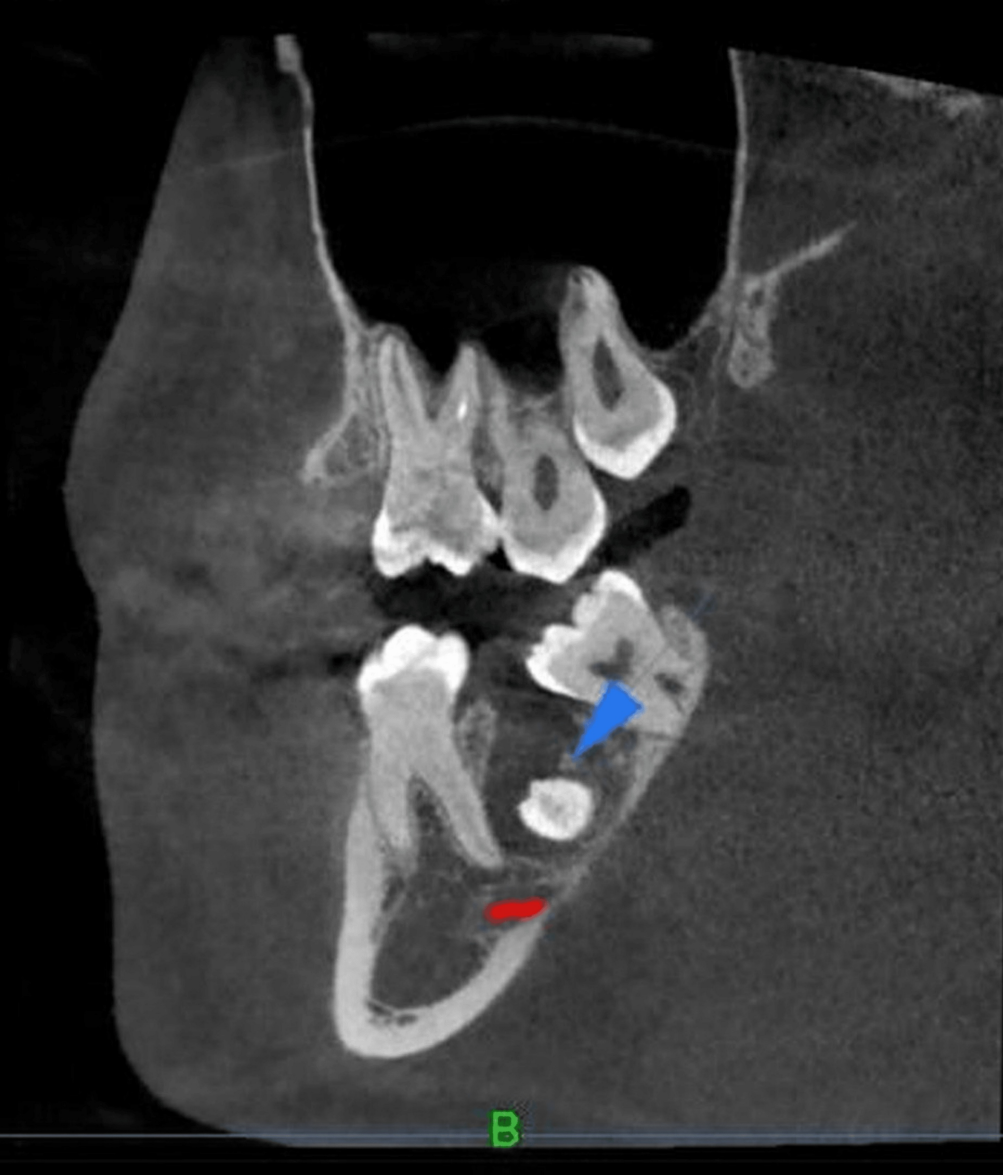
CBCT view showing impacted 47 with hypodense lesion Cone-Beam Computed Tomography (CBCT) sagittal section showing an impacted mandibular right second molar (47) associated with a well-defined pericoronal hypodense area. A small focal speck of internal radiopacity is noted within the hypodensity (blue arrow), with the lesion confined to the coronal aspect of the impacted tooth 47. CBCT was used for three‑dimensional assessment as recommended for precise localization of odontogenic lesions.

In CBCT, a well-defined hypodensity with a surrounding hyperdense corticated border was noted, measuring approximately 10*11mm in size, enclosing the crown of impacted 47 with a speck of hyperdensity within the lesion, with close approximation to the superior cortex of the inferior alveolar nerve canal (Figure 3). Despite the close proximity of the impacted tooth (47) to the mandibular canal, the patient did not report any numbness, tingling, or altered sensation in the inferior alveolar nerve distribution. Radiographic differential was given as dentigerous cyst, odontogenic keratocyst, unicystic ameloblastoma for the reasons of impacted tooth, posterior region and radiolucent lesion. For a radiopacity within the radiolucency differential include COF, odontogenic-myxoma and calcifying odontogenic tumor.

TMJ MRI suggested disc displacement with reduction in right side and without reduction in left side. Thus, imaging investigations identified two separate conditions co-existing in the same patient: TMJ internal derangement confirmed through MRI and an incidental mixed radio-dense lesion detected through CBCT. Thus according to Kamar Alden et al., jaw locking and deviation may coexist with odontogenic pathology and warrant concurrent temporomandibular joint disorder (TMD) assessment in young adults.

**Figure 4:**
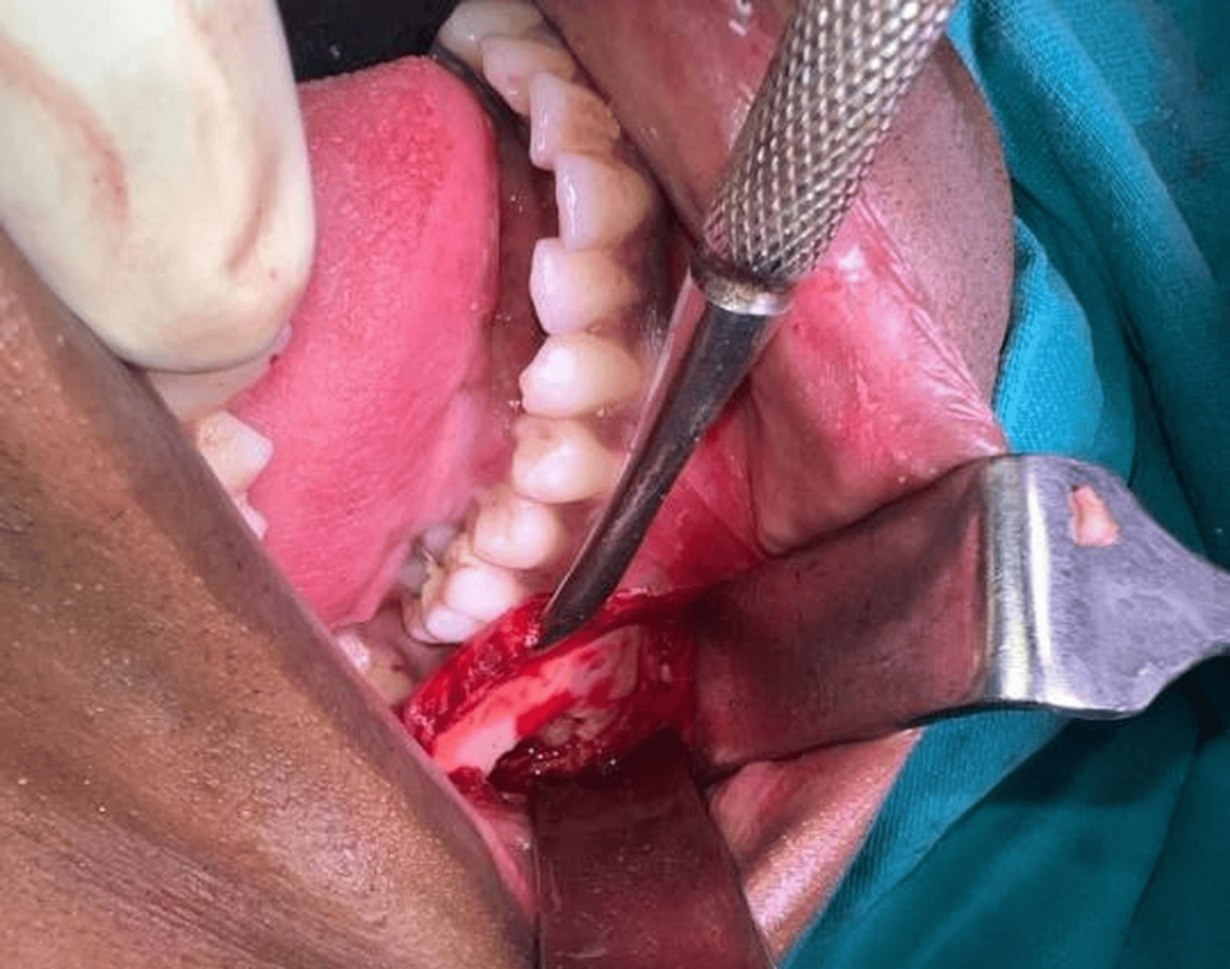
Surgical excision under general anesthesia Intraoperative clinical photograph showing surgical exposure and removal of the impacted 47 along with en bloc excision of the associated lesion.

For the TMJ internal derangement, conservative therapy was initiated, including pharmacologic management comprising analgesics and muscle relaxants followed by lower occlusal splint therapy. The patient was placed on regular follow-up and after symptomatic improvement in TMJ function we opted for management of the impacted 47. Surgical excision along with extraction of impacted 47,48 was done under general anesthesia (Figure 4) and given for histopathological examination.

**Figure 5:**
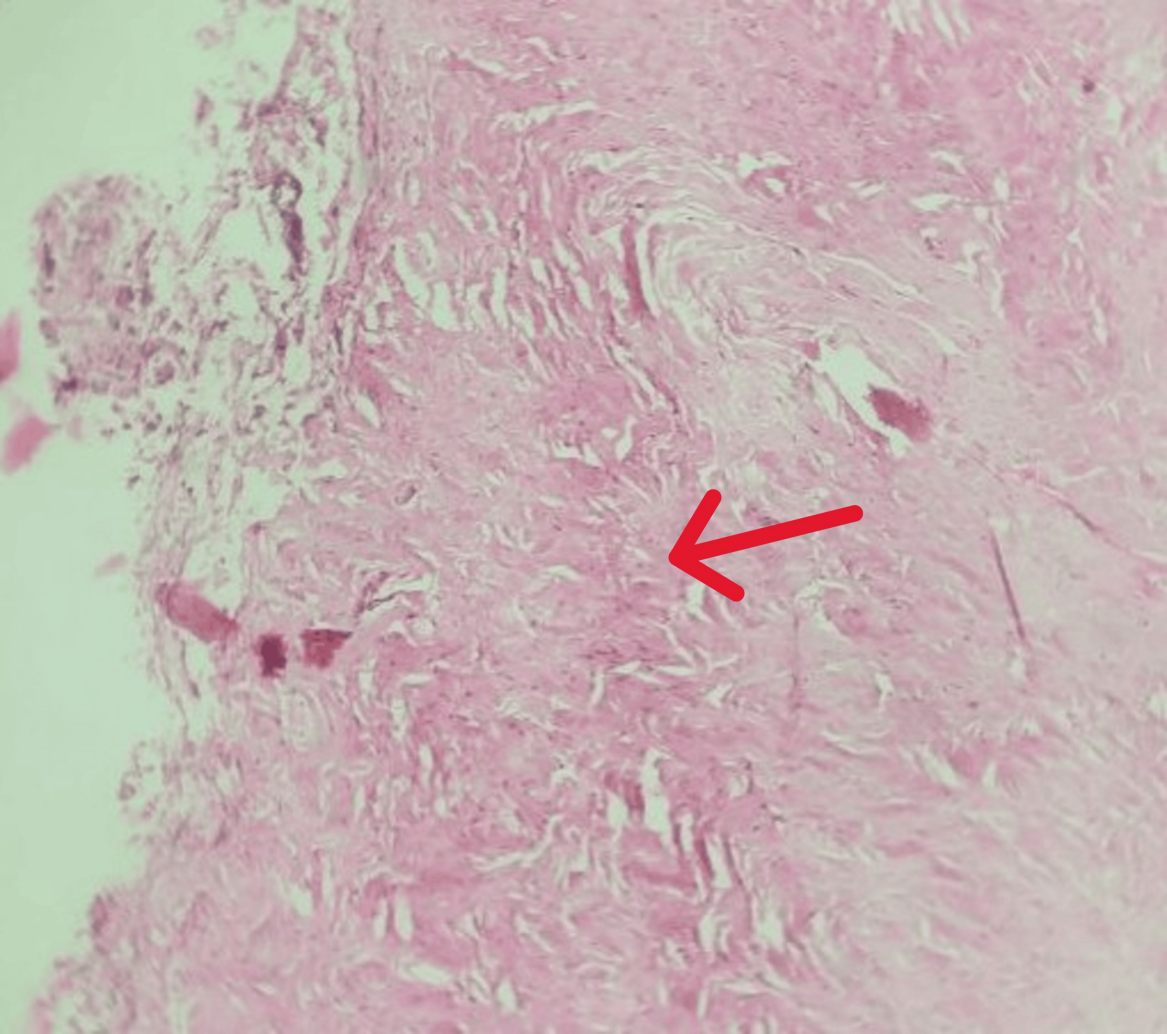
Histopathological image showing areas of dense bundles of collagen fibers. Histopathological section (Hematoxylin and Eosin stain, low-power magnification) showing a moderately cellular fibrous connective tissue stroma composed of interlacing bundles of collagen fibers (red arrow) with scattered spindle-shaped fibroblasts.

The histopathology (Figure 5) shows a fibrous connective tissue stroma composed of dense interwoven collagen bundles with pronounced hyalinization. The fibroblast cells are sparse and slender, and discrete areas of calcification characterized by dentinoid matrix are seen, which is suggestive of odontogenic fibroma.

**Figure 6:**
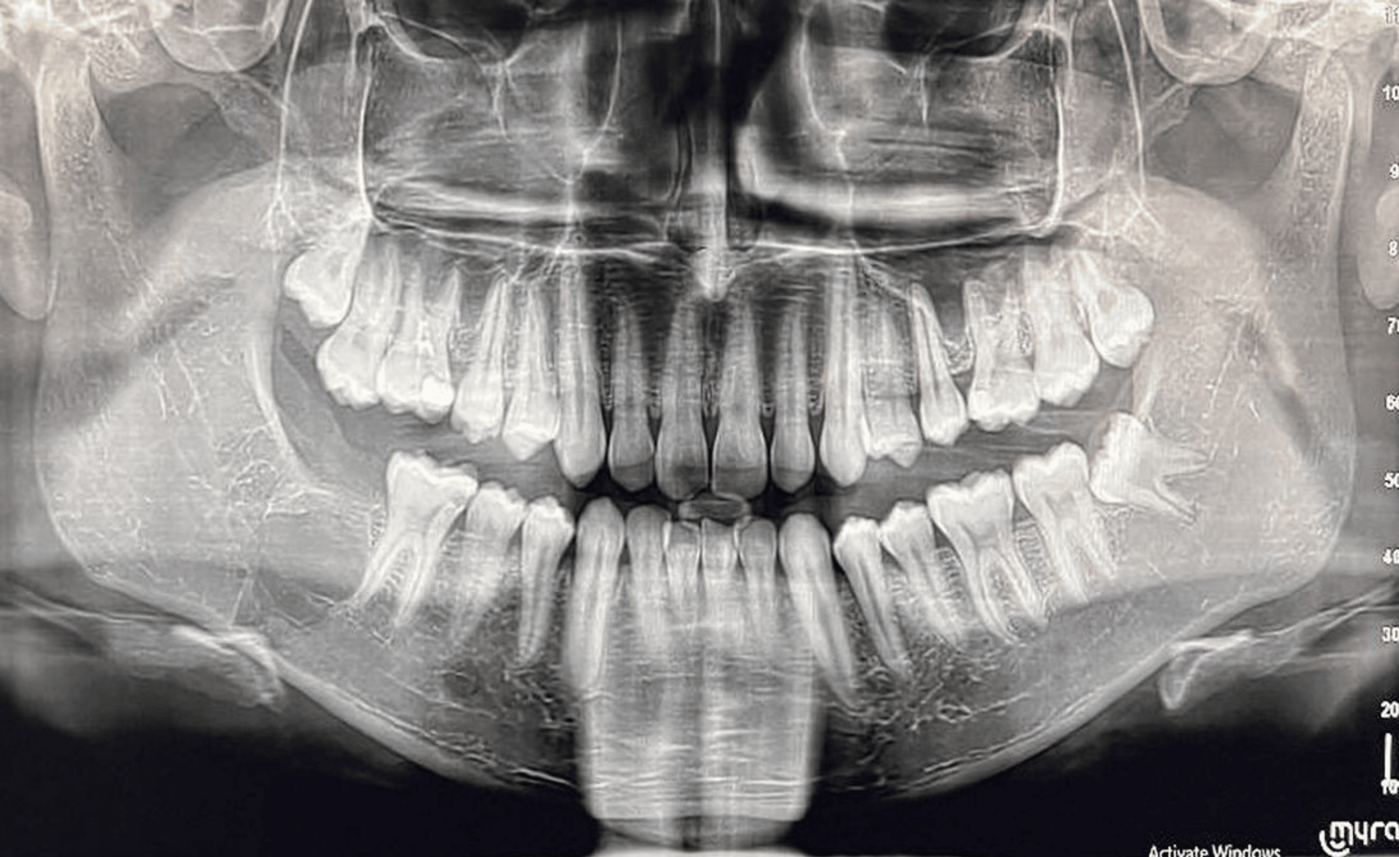
At the one-month follow-up visit, radiographic findings were within normal limits, consistent with satisfactory postoperative healing. Postoperative orthopantomogram demonstrating complete removal of the impacted 47,48 and the associated lesion, with no residual radiolucency evident at the surgical site.

The patient showed marked improvement in temporomandibular disorder symptoms after medication and occlusal splint therapy. At the one-month follow-up visit, radiographic findings were within normal limits, consistent with satisfactory postoperative healing (Figure 6). There was no neurosensory deficit noted clinically both before and after surgical intervention. The patient continues under periodic follow-up and to date, there has been no evidence of lesion recurrence.

## Discussion

Odontogenic fibroma is a rare benign fibroblastic neoplasm, which may contain inactive odontogenic epithelium with or without calcifications [1]. According to the 2022 WHO classification, COF is categorized under benign mesenchymal odontogenic tumors [1]. Its rarity is underscored by reports indicating that it accounts for approximately 0.1% of all central odontogenic tumors, making it an uncommon diagnostic consideration in routine clinical practice [3]. WHO and Gardner in 1980 recognized two types of lesion, a simple central odontogenic fibroma and a central odontogenic fibroma which was again revised in 2005 by Gardner and classified as (1) the WHO variant (containing odontogenic epithelium) and (2) the non-WHO variant (without odontogenic epithelium) [4]. Although reported across a wide age spectrum, it most frequently occurs between the second and fourth decades. A female predilection is commonly reported [4,5]. The most common site of involvement is the maxillary anterior region and the mandibular molar region [6]. Clinically, the lesion commonly manifests as a painless swelling but sometimes it presents with facial asymmetry, tooth mobility, trismus, tooth displacement and delayed eruption [7].

Wesley et al.'s diagnostic criteria (1975) [9] are stated as follows: First criteria pertains to clinical features, where the lesion is in the center of the jaws and has a slow persistent growth resulting in painless expansion. The second criteria pertains to radiographic feature, where unilocular or multilocular radiolucent lesions sometimes associated with unerupted or displaced teeth. The third criteria pertains to histopathological findings, where predominantly mature collagen fibers with numerous fibroblasts and the presence of small nests and/or strands of inactive odontogenic epithelium which is a variable feature and the fourth criteria suggests, since it is a benign condition, it responds well to surgical enucleation with no tendency to recur. Based on topographic location, two variants are recognized: an extraosseous (peripheral) odontogenic fibroma (POF) and an intraosseous (central) odontogenic fibroma (COF) [10].

Likewise, the present case demonstrated pain localized to the temporomandibular joint region rather than the lesion site, suggesting that the symptom was unrelated to the odontogenic fibroma, since COF appears as an asymptomatic condition. Additionally, the lesion was associated with an impacted mandibular molar, a feature that has been consistently reported in the literature. COF is associated with an unerupted tooth in one‐third of the cases [11]. Radiographically, the lesion presented as a well-defined radiolucency with a speck of radiopacity, deviating from the classic purely radiolucent appearance, but consistent with some cases showing calcified foci within the fibrous stroma. Radiographically it appears unilocular or multilocular radiolucency with well-defined borders. Smaller lesions typically present as unilocular radiolucencies, whereas larger lesions may exhibit scalloped margins or a multilocular appearance [12]. Cortical expansion is commonly observed in extensive lesions, as seen in the present case; however, cortical perforation is not a characteristic feature. The lesion margins are generally well defined, circumscribed, or well demarcated, often without an associated sclerotic border. The bony septa forming the locules are usually thin and may not appear prominently radiopaque. In some cases, scattered radiopaque flecks may be present, and in rare instances, the lesion may appear as a homogeneous radiopaque mass [12].

Based on the initial clinical and radiographic findings, several odontogenic entities were considered in the differential diagnosis which includes dentigerous cyst, ameloblastoma and ameloblastic fibroma, odontogenic-myxoma, and calcifying odontogenic tumor. Other differentials include adenomatoid odontogenic tumor, complex odontoma, fibrosarcoma, globulomaxillary cyst, and lateral periodontal cyst [12]. Ultrastructural observations suggest that central odontogenic fibroma and odontogenic myxoma exhibit considerable morphological overlap and appear to share a similar histogenetic origin [13]. However the histopathological examination revealed characteristic features of a mature collagenous stroma with sparse spindle-shaped fibroblasts and discrete areas of calcification, thereby establishing the definitive diagnosis of odontogenic fibroma and excluding other differential diagnoses.

Surgical excision remains the treatment of choice for COF and was successfully performed in the present case. The tumor is considered non-aggressive, and recurrence is uncommon [14]. Reported recurrence rates are approximately 6%, with an annual recurrence rate of 1.4% [2], emphasizing the favorable prognosis associated with adequate surgical management. Nonetheless, long-term follow-up is recommended due to the lesion’s rarity and occasional reports of recurrence.

## Conclusions

Central odontogenic fibroma is a rare benign non-aggressive odontogenic tumor often presenting with non-specific clinical and radiographic features that mimic other fibro-osseous lesions and odontogenic lesions. Early recognition is crucial as timely surgical enucleation and curettage offers the excellent prognosis with minimal risk of recurrence. Overall this report underscores the need for clinicians to consider this in the differential diagnosis of mixed lesions of the jaw, enabling appropriate treatment and improved patient outcome. This case highlights the importance of correlating clinical, radiographic and histopathological findings to arrive at an accurate diagnosis, particularly because this can resemble more common jaw lesions. A multidisciplinary approach ensured successful management, and the patient demonstrated uneventful healing with no evidence of recurrence during follow-up.
